# Recent Advances of Nanotechnology-Facilitated Bacteria-Based Drug and Gene Delivery Systems for Cancer Treatment

**DOI:** 10.3390/pharmaceutics13070940

**Published:** 2021-06-24

**Authors:** Chaojie Zhu, Zhiheng Ji, Junkai Ma, Zhijie Ding, Jie Shen, Qiwen Wang

**Affiliations:** 1 Department of Cardiology, The First Affiliated Hospital, Zhejiang University School of Medicine, Hangzhou 310003, China; 3180101530@zju.edu.cn; 2 Chu Kochen Honors College of Zhejiang University, Hangzhou 310058, China; 3180103158@zju.edu.cn (Z.J.); 3180101531@zju.edu.cn (J.M.); 3 Institute of Pharmaceutics, College of Pharmaceutical Sciences, Zhejiang University, Hangzhou 310058, China; 4 College of Letters & Science, University of California, Berkeley, CA 94704, USA; jerryding2022@berkeley.edu; 5 Department of Pharmacy, School of Medicine, Zhejiang University City College, Hangzhou 310015, China

**Keywords:** bacterial therapy, nanotechnology, drug and gene, delivery system, combinational therapy, cancer treatment

## Abstract

Cancer is one of the most devastating and ubiquitous human diseases. Conventional therapies like chemotherapy and radiotherapy are the most widely used cancer treatments. Despite the notable therapeutic improvements that these measures achieve, disappointing therapeutic outcome and cancer reoccurrence commonly following these therapies demonstrate the need for better alternatives. Among them, bacterial therapy has proven to be effective in its intrinsic cancer targeting ability and various therapeutic mechanisms that can be further bolstered by nanotechnology. In this review, we will discuss recent advances of nanotechnology-facilitated bacteria-based drug and gene delivery systems in cancer treatment. Therapeutic mechanisms of these hybrid nanoformulations are highlighted to provide an up-to-date understanding of this emerging field.

## 1. Introduction

**Figure 1:**
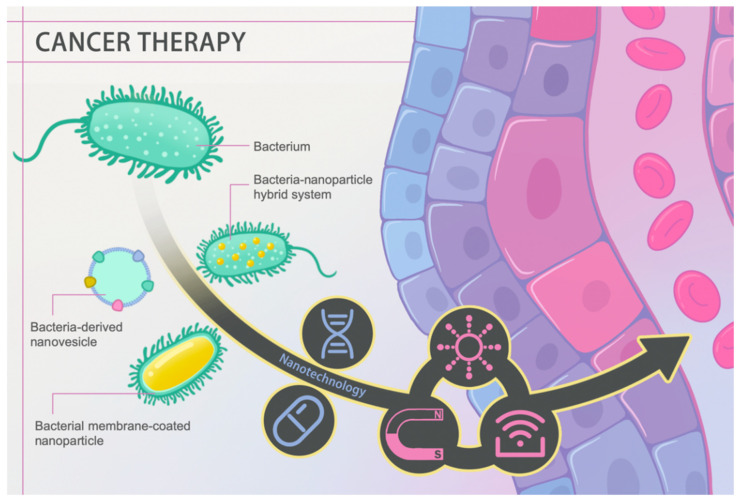
Schematic diagram of nanotechnology-facilitated bacteria-based cancer therapy. Bacteria-derived nanovesicles, bacterial membrane-coated nanoparticles, and bacteria–nanoparticle hybrid systems represent the three main representative delivery platforms used to date. Such platforms not only facilitate drug/gene loading and delivery, but they can also perform diverse functions in response to external stimuli, such as light, magnetism, and ultrasound, achieving better therapeutic efficacy.

Malignant tumors are the second most common threat to human life and health [1]. Countless efforts have been dedicated to countering tumor growth and rapidly progressing associated diseases. Currently, conventional clinical interventions like chemotherapy still face problems such as off-target toxicity, limited therapeutic agent enrichment in target lesions, and drug resistance despite being the first-line clinical treatment against cancer [2]. Certain bacteria exhibit promising properties in handling these defects. In 1813, Vautier found that those suffering from cancer had their condition improved after the development of gas gangrene. The underlying therapeutic efficacy is mainly attributed to the ability to localize a hypoxic environment, toxin release, and immune activation using pathogenic bacteria [3]. In recent years, advancing nanotechnology has extended bacterial therapies to a higher level through tailoring bacteria on a nanoscale, such as bacteria-derived nanovesicles and bacterial membrane-coated nanoparticles, or endowing bacteria with abilities to serve as drug carriers, photosensitizers, and sonosensitizers (Figure 1). In this review, cancer hallmarks, current management regimens and their deficiencies are first introduced. Then, we elaborate on the history and therapeutic mechanisms of conventional bacterial therapy. Most importantly, we highlight recent advances in nanotechnology-facilitated bacterial therapy. The superiority of such hybrid nanoformulations, either performing as drug and gene delivery vectors or active pharmaceuticals themselves, is described in detail. Overall, rapidly advancing nanotechnology has facilitated bacterial therapy, unlocking a new stage in cancer treatment.

## 2. Cancer Hallmarks and Targeted Therapy

Cancer was first described in the Edwin Smith and Ebers Papyri approximately between 1500 and 1600 BC. In particular, the Edwin Smith Papyrus provided the first description of breast cancer, in which breast cancer was described as cool to the touch, bulging, and believed to be undefeatable [4].

Later in the 20th century, various treatments have been put forth to deal with such disease, most notably chemotherapy [5]. Chemotherapy conventionally utilizes chemotherapeutic drugs like cyclophosphamide in their free forms to kill the tumor [6]. However, such small molecular agents also exert cytotoxic impact on noncancerous cells. In this case, systematic toxicity, which manifests as fatigue, nausea, and blood disorders, has aroused intensive public concern. Moreover, high liposolubility of certain agents like paclitaxel brings difficulties to their intravenous administration [7]. In addition, several molecules like camptothecin are unstable in physiological condition and may be inactivated before being taken up by cancer cells, which severely hampers their therapeutic effects [8]. Therefore, a more comprehensive understanding of cancer characteristics is urgently required to come up with innovative therapies with higher on-target toxicity and inert properties toward normal tissues.

**Figure 2:**
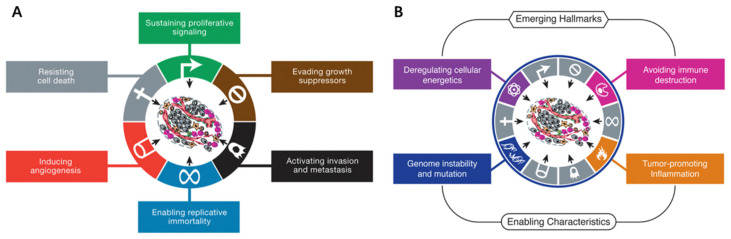
(**A**) The six hallmark traits originally proposed in 2000. The past decade witnessed remarkable progress toward understanding the mechanistic underpinnings of each hallmark. (**B**) Emerging Hallmarks and Enabling Characteristics. Reproduced with permission from [10], 2011, Elsevier.

In 2000, Douglas Hanahan and Robert A. Weinberg summarized six major traits of human cancer collectively named ‘cancer hallmarks’: evading apoptosis, self-sufficiency in growth signals, insensitivity to anti-growth signals, tissue invasion and metastasis, limitless replicative potential, and sustained angiogenesis [9]. Ten years later, the reprogramming of energy metabolism, evading immune detection, genomic instability, and tumor-promoting inflammation were added as additional characteristics (Figure 2) [10]. Essentially, the occurrence of cancer is rooted in genetic mutation(s), which is attributed to environmental risk factors like viral infection and behavioral risk factors such as smoking [11].

These summarized cancer hallmarks provide significant guidance to innovate novel therapies against cancer. For example, the term ‘targeted therapy’ has been suggested and obtained enormous expectations due to superior specificity and therapeutic mechanism. Conventionally, targeted therapy uses therapeutic agents to target specific genes or proteins associated with cancer hallmarks such as tumor cell growth and proliferation. Such therapy can be broadly classified into antibody-based and small molecule-based therapies [12]. Antibody-based therapy acts through specific binding to the proteins present on target tumor cells, for example, human epidermal growth factor receptor 2 (HER2) on human breast cancer cells [13]. Small molecules, including multikinase small molecule inhibitors and selective small molecule inhibitors, act by inhibiting the kinase or cytokine to block certain signaling pathways. For example, pirfenidone can inhibit p38γ, a mitogen-activated protein kinase, to reduce cutaneous T cell lymphoma cell viability [14]. Compared to chemotherapy which acts by killing both cancer cells and normal cells, targeted therapy takes effect on cancer cells more precisely and has achieved significant advancements [15]. For example, the response rate to imatinib for treating chronic myeloid leukemia is 90% compared with 35% achieved with conventional chemotherapy [16]. In addition, gene therapy has similarly realized notable improvements. Such therapy takes effect through up/downregulating the expression of specific genes and proteins and has brought promising clinical outcomes [17].

Regardless of the improved therapeutic efficacy that targeted therapies and gene therapies have achieved, drug resistance and deficiency in on-target agent delivery severely hinders their potential in clinical cancer treatment [18]. Especially for gene therapy, the problem of rapid degradation and short half-life needs to be solved before their full potential is realized in treating cancer. An appropriate therapeutic agent possessing tumor-targeting abilities and therapeutic measures, such as used bacterial systems, might simultaneously achieve these goals of modern cancer treatment.

## 3. Bacteria, an Old Player against Cancer

Bacteria are some of the most notorious killers in human history. In the 14th century, the Black Death claimed millions of human lives, which was caused by the bacterium *Yersinia pestis* [19]. However, bacteria also contain promising antitumor properties beneath their ‘evil masks’. In this section, we elaborate on the history (Table 1) and therapeutic mechanisms of bacterial therapy. Bottlenecks confronted in clinical trials are also mentioned to acquire a comprehensive understanding of the current role of bacteria in cancer treatment at the same time.

### 3.1. Development of Bacterial Therapy against Cancer

In 1891, William B. Coley inoculated *Streptococcus pyogenes* for the treatment of malignant sarcoma, thus becoming the pioneer of bacterial therapy [21]. Regardless of its excellent curative effect, the risks induced by bacterial infection could not be neglected. To improve the feasibility and reduce the latent infection, Coley inactivated *Streptococcus pyogenes* and *Serratia marcescens* to fabricate the famous bacterial formulation ‘Coley’s toxins’ [27]. However, due to individual disparity, poor replicability in clinical treatment, and the rise of radiotherapy and chemotherapy in the early 20th century, bacterial therapy remained stagnant until it was revisited in the 1990s.

In 1989, the first bacteria-based formulation, the Bacillus Calmette–Guerin vaccine (BCG) was approved by the FDA for the clinical treatment of bladder cancer [22]. Later, the underlying therapeutic mechanisms were gradually disclosed. For example, after intravesical instillation of BCG, a series of immune cascade reactions are triggered locally, stimulating immune cells to secrete TNF-α, IL-12, and other factors to promote tumor cell apoptosis [28,29].

After that, various innovative bacteria-based cancer therapies underwent passionate investigations. In addition to traditional detoxification and inactivation pretreatment, genetically engineered bacteria exhibit promising therapeutic capability against cancer. With the aid of genetic modifications, the toxicity of bacteria can be reduced and the selective targeting capabilities are greatly enhanced [30]. For example, a strain of *Salmonella typhimurium* (VNP20009) which is genetically modified through chromosomal deletion of the purI and msbB genes reduced the virulence and the risk of septic shock [23]. Genetically modified leucine–arginine-deficient *Salmonella typhimurium* A139 possesses unprecedented tumor-targeting ability [31].

In recent years, the development of synthetic biology provided strong theoretical and technical support for further optimization of bacterial therapy. The design of the logic-gates system, kill switch, quorum sensing, and other genetic pathways reprogrammed bacteria and endowed them with diverse diagnostic and therapeutic superiorities, such as the capabilities of sensing external changes, responding to environmental alterations, tumor targeting, and selective toxicity to certain cells. Such emerging fields have recently been extensively summarized elsewhere [25,32,33]. In this review, we focus on the advantages that nanotechnology brings to bacterial therapy.

### 3.2. Main Mechanisms of Bacterial Therapy

#### 3.2.1. Tumor-Targeting Mechanisms

Currently, two main mechanisms explain the tumor-targeting ability of bacteria, which are high hypoxia and immunosuppression in the tumor microenvironment. When some anaerobic bacteria, for example, *Salmonella*, were injected intravenously into mice, there was no significant difference in the amount of bacteria between the tumor and the liver at the beginning. Subsequently, the bacteria localized near the tumor proliferated due to a suitable hypoxic environment and immunosuppressed conditions [34]. In addition, those situated at normal tissues or in the body’s circulation were rapidly eliminated due to natural immune clearance [35].

In addition, interstitial fluid pressure (IFP) has been reported to be higher in tumor tissues due to blood vessel leakiness and poor lymphoid fluid drainage [36]. As a result, such increased tumor IFP hinders conventional therapeutic agents from entering deep tumor tissues, thus impacting their uptake by cancer cells. The flagellum of bacteria can well handle this predicament through active migration toward tumor tissues and even deeper into their necrotic core [37]. Other factors like the entrapment of bacteria in chaotic tumor vasculatures and chemotaxis toward compounds that derive from cancer tissues also contribute to their tumor-targeting ability [38,39,40].

#### 3.2.2. Therapeutic Mechanisms

The therapeutic mechanisms of bacteria can be classified into three groups: (1) swelling and apoptosis of tumor cells induced by bacterial invasion, (2) secretion of bacterial toxins, and (3) antitumor immune activation. First, bacteria can kill tumor cells by initiating autophagy or inducing cell apoptosis through infection and intracellular multiplications [41]. In addition, bacteria can secrete toxins which can activate downstream apoptotic pathways. For example, cytolysin A (ClyA) can trigger caspase-mediated cell death and form gaps in cell membranes [42]. *Escherichia coli* K-12 can secrete ClyA and inhibit tumor growth. Besides, nitric oxide (NO) correlates with tumor progression. A high concentration of NO has been reported to mediate cancer cell apoptosis and tumor regression [43]. However, under normal conditions, NO is converted to its nontoxic form NO^3−^. In this case, the NO generation enzyme produced by *E. coli* reoxidizes NO^3−^ into NO to block cancer progression [44].

Apart from them, therapeutic effects rely on the antitumor immune responses to a large extent. Bacteria exhibit outstanding immune activation capability. For example, *Salmonella* can colonize macrophages and dendritic cells to induce the production of interleukin-1β (IL-1β) [45]. *Salmonella* infection can also lead to the upregulation of connexin 43 (Cx43) and the formation of functional gap junctions between dendritic cells and tumor cells [46]. Such junctions assist tumor-associated antigens being presented to T cells from dendritic cells, resulting in significant antitumor immune responses. In addition, pathogen-associated molecular patterns (PAMPs) show the capability to activate inflammatory responses and facilitate proinflammatory cytokine release which can contribute to cancer immunotherapy [47]. For example, lipopolysaccharide (LPS) can induce toll-like receptor 4 (TLR4) signal transduction and promote macrophage secretion of IL-1β [48]. Flagellin is also a potential stimulator of natural killer cells that can induce the production of interferon-γ [49,50].

## 4. A New Role for the Old Player

As previously mentioned, bacteria exhibit outstanding antitumor curative capabilities due to their tumor-targeting ability and various therapeutic mechanisms which include induction of tumor cell lysis and activating antitumor immune responses. However, such an excellent antitumor agent still confronts many bottlenecks like potential toxicity to normal tissues, latent inflammation, and the inability of monobacterial therapy to fully eradicate established tumors [51]. In recent years, nanotechnology has achieved tremendous progress in biomedicine, especially in cancer treatment. For example, Mahwash Mukhtar et al. have summarized the recent advances of nanomaterials achieved in treating brain cancer [52]. They disclosed the unique advantage of blood–brain barrier permeation ability of nanomaterials, which facilitates brain lesion delivery of therapeutic agents. Nanotechnology helps mobilize substances at the nanoscale, which endows them with various fascinating properties, such as highly efficient drug loading, elevated tumor targeting ability, and other functions like photosensitization and novel catalytic activity [53]. Numerous innovative cancer therapies have been proposed based on nanomaterials with different properties, such as photodynamic, photothermal, magnetic heat, and immune therapies [54,55,56]. In this section, we elaborated on how nanotechnology facilitates bacterial therapy against cancer including bacterial membrane-based nanoformulations (including bacteria-derived nanovesicles and bacterial membrane-coated nanoparticles), bacteria–nanoparticle hybrid drug and gene delivery systems, and functional bacteria–nanoparticle hybrid platforms. Therapeutic mechanisms and superiorities are especially highlighted to acquire a deep understanding of the synergistic effect of nanotechnology and bacteria.

### 4.1. Bacterial Membrane-Based Nanoformulations against Cancer

In the following section, bacterial membrane-based nanoformulations are discussed based on the structure of the platform, including bacteria-derived nanovesicles and bacterial membrane-coated nanoparticles.

#### 4.1.1. Bacteria-Derived Nanovesicles as Drug and Gene Delivery Systems

Bacteria-derived nanovesicles (BDNVs) are composed of a double lipid layer with a size range of 20–400 nm. BDNVs are mainly classified into four groups, outer membrane vesicles (OMVs), outer–inner membrane vesicles (OIMVs), double-layered membrane vesicles (DMVs), and cytoplasmic membrane vesicles (CMVs), based on their structures and sources [57,58,59]. Various synthetic methods have been suggested. For example, OMVs can be derived through bacteria blebbing [60]. OMVs can be obtained by explosive cell lysis [61]. These types of vesicles are virtually the same, only with several main molecules varying between different species. For example, outer membranes are composed of lipopolysaccharide, while cytoplasmic membranes exhibit lipoteichoic acid on their surfaces [62,63,64].

Over the recent years, bacteria-derived nanovesicles have been exploited against cancer progression. With their nanoscale size, bacteria-derived nanovesicles possess tumor penetration ability with the feasibility of surface modification and improved drug loading capacity (Table 1). For example, Jennifer MacDiarmid et al. derived 400-nm nanovesicles from several genetically modified bacteria such as *Escherichia coli* which can be loaded with chemotherapeutic agents like doxorubicin to effectively treat cancer [65]. In their study, they further modified this nanovesicle with an epidermal growth factor receptor (EGFR) antibody through bispecific antibodies to target breast cancer. As a result, 30% of the total EGFR-targeted nanovesicles reached the tumor site, approximately 20 times more compared to nonmodified nanovesicles. Furthermore, a 100-fold higher dose (100 μg) of doxorubicin is required to match the therapeutic effect of liposomal doxorubicin (1 μg) in the form of EGFR-targeted nanovesicles. In their later research, they even proved the feasibility of using such bacteria-derived nanoplatforms to deliver siRNAs for drug-resistant tumor treatment [66]. In the said study, they exploited a dual sequential therapeutic strategy by first knocking down drug resistance-related proteins through siRNA delivery and sequential chemotherapeutic agent delivery based on these nanovesicles. As a result, this delivery nanoplatform, combined with the dual sequential strategy, resulted in 100% survival up to 110 days after xenografting of MES-SA/Dx5 human uterine cells, an aggressive multidrug-resistant tumor cell line. In conclusion, such bacteria-derived nanovesicles exhibit excellent drug- and gene-carrying capability. Further work is needed to investigate other chemotherapeutic agents and gene-based drug-carrying capability of this nanoplatform to extend its therapeutic usage. The biodistribution of this nanoplatform also needs to be intensively investigated to ensure its validity toward other cancer types.

#### 4.1.2. Other Functional Properties of Bacteria-Derived Nanovesicles

Apart from their drug- or gene-carrying capacity, bacteria-derived nanovesicles also possess the capability of activating immune responses to treat cancer. Bacterial OMVs composed of diverse immunostimulatory molecules have recently been investigated for vaccine and delivery system use. For example, Kim et al. demonstrated the antitumor potential of OMVs derived from genetically modified *Escherichia coli* [67]. In their study, OMVs exhibited excellent tumor-targeting ability due to their enhanced permeability and retention (EPR). Such immunomodulatory agents could efficiently induce the production of antitumor cytokines such as CXCL10 and interferon-γ. Together, these immune nano-stimulators successfully eradicated established tumors and rejected tumor rechallenges.

To take the technology further, Qing et al. recently reported an OMV-based tumor microenvironment ‘reprogrammer’ [68]. In their study, they chemically modified OMVs derived from *Escherichia coli* BL21 cells with calcium phosphate (CaP) shells. As a result, CaP shells, the pH-sensitive shields, not only assisted the avoidance of severe systemic inflammation which could be potentially induced by naked OMVs, but also neutralized the acidic tumor microenvironment to polarize tumor-associated macrophages from the proinflammatory M1 phenotype to the anti-inflammatory M2 phenotype, which synergized with the intrinsic immunostimulatory effect of OMVs and eventually led to 60% survival rate at day 80 compared with 0 in the group applying naked OMVs.

**Figure 3:**
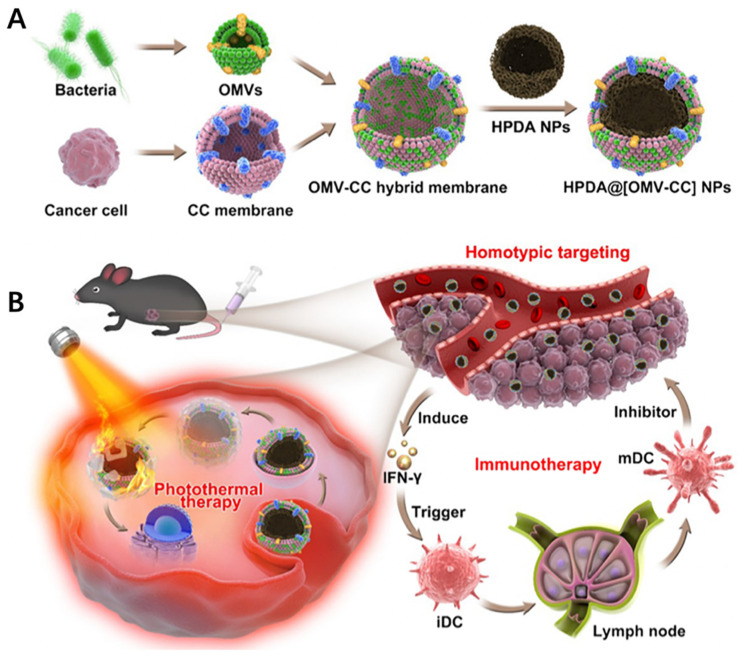
(**A**) Schematic of the membrane derived from OMV and CC fusion and the resulting fused membrane camouflaged HPDA NPs to produce HPDA@[OMV-CC] NPs. (**B**) Synergistic photothermal/immunotherapy of melanoma. Reproduced with permission from [60], American Chemical Society, 2020.

In another approach, nanoparticles can also be inserted into bacteria-derived nanovesicles to provide additional functions like photosensitivity. For example, Wang et al. recently developed bacteria–cancer cell hybrid membrane-coated photosensitizing hollow polydopamine nanoparticles (HPDA@[OMV–CC]) (Figure 3A) [69]. In this study, bacterial membranes effectively induced the production of antitumor cytokines through various immunostimulatory membrane components. Cancer cell membranes served as the source of tumor antigen, which synergized with antitumor cytokines to induce significant immune responses against cancer. As a result, the combination of photothermal treatment and cancer immune therapy successfully resulted in complete eradication of melanoma (Figure 3B).

Insertion of nanoparticles into bacterial membranes not only confers platforms with added functionality like photothermal responses as mentioned above, but could also help enhance their ability to induce immune responses to fight against cancer. For example, Patel et al. developed bacterial membrane-coated nanoparticles (BNPs) composed of the PC7A/CpG polyplex core that was functionalized with imide groups [70]. In their study, radiation was first applied to stimulate cancer cells to release neoantigens. BNPs were then injected intratumorally. As a result, the imide groups on the surface of BNPs assisted the sequestration of the neoantigens resulting from radiation. The inner core component, CpG, accelerated the maturation of antigen-presenting cells. PC7A, a pH-responsive polymer, facilitated endosomal escape and antigen cross-presentation. This nanoplatform facilitated the in situ immunorecognition of radiation-treated tumors, resulting in remarkable tumor regression and long-term antitumor immune memory. Most importantly, it provides a personalized approach to cancer immunotherapy.

To summarize the above, drug or gene loading and immune modulation are the major uses of bacteria-derived nanovesicles. Chemical functionalization, antibody conjugation, genetic modification, and functional nanocore insertion are several of the main measurements to enhance targeting ability and therapeutic efficacy (Table 2). However, several critical issues need to be thoroughly investigated before their potential translation into clinical use. Firstly, excessive amounts of ingredients are applied in the synthetic procedure. The metabolic pathway of these components needs to be well-understood to ensure their biosafety. Secondly, work that verifies the replicability of such complex therapeutic nanoplatforms’ therapeutic efficacy needs to be carried out to guarantee their validity in cancer treatment.

### 4.2. Bacteria–Nanoparticle Hybrid System

In this section, we elaborated in detail on the current knowledge of the bacteria–nanoparticle hybrid drug and gene delivery systems in treating cancer. The therapeutic mechanism and superiorities are highlighted. Nanoparticles, which act as functional agents in this hybrid system, such as photosensitizers and catalysts, are also discussed to extend the understanding of the biomedical potential of such hybrid systems.

#### 4.2.1. Drug and Gene Delivery

**Figure 4:**
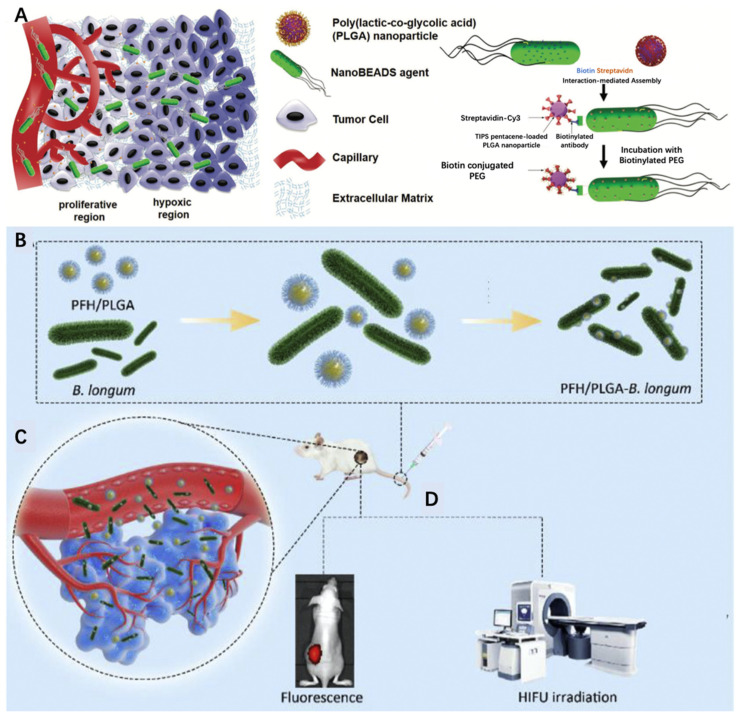
(**A**) Schematic illustrating enhanced penetration of NanoBEADS in a poorly vascularized tumor tissue compared with passively diffusing nanoparticles. Reproduced with permission [78], 2018, Wiley-VCH GmbH, Weinheim. (**B**) Synthesis of PFH/PLGA–*Bifidobacterium longum*. (**C**) Targeting the tumor tissue. (**D**) Fluorescence imaging and HIFU therapy. Reproduced with permission [79], 2019, Elsevier.

Although nanoparticles have achieved great advancements in drug carriage to treat cancer, multiple barriers hinder their enrichment in tumor tissues such as interstitial fluid pressure and extracellular matrix blockage. With the help of active targeting provided by bacteria migration and high drug-loading efficiency realized through nanoparticle carriage, such hybrid delivery systems have achieved great advancements in the target delivery of therapeutic agents. For example, Suh et al. developed a bacteria-enabled autonomous drug delivery system (NanoBEADs) [78]. In their study, they combined poly(lactic-co-glycolic acid) (PLGA) nanoparticles with the *S. typhimurium* VNP20009 bacterium through the streptavidin–biotin interaction (Figure 4A). As a result, this conjugation had no impact on the tumor penetration and the targeting of bacteria. PLGA nanoparticles achieved 100-fold increased enrichment in the tumor compared to their passively diffusing counterparts. Further efforts should be dedicated to the verification of the therapeutic efficacy of this delivery platform. For example, Luo et al. recently innovated a hybridized platform to realize high-intensity focused ultrasound (HIFU) therapy against cancer. In their research, they conjugated perfluorohexane (PHF)-loaded PLGA nanoparticles onto *Bifidobacterium*, which exhibited great tumor-targeting ability and thereby improved therapeutic and diagnostic efficacy (Figure 4B–D) [79].

Aside from this, gene therapy can also be realized through bacteria–nanoparticle hybrid delivery systems. Effective gene delivery needs to overcome several barriers, including in terms of protection against endogenous nuclease degradation, cellular uptake elevation, and endosomal avoidance [80,81]. However, conventional gene delivery nanoplatforms, such as mesoporous silica nanoparticles, are unable to transfer nucleic acid into host cells due to the failure of endosomal escape [82]. In this case, bacteria exhibit excellent gene delivery capabilities. For example, *Listeria monocytogenes* can escape from intracellular vesicles via the pore formation activity of listeriolysin O. After endosomal escape, the loaded genes can diffuse to the nucleus for plasmid DNA and the cytoplasm for siRNA to implement their mission. Akin et al. innovated a bacteria–nanoparticle hybrid delivery system for efficient drug and gene delivery into tumor cells [83]. In their investigation, nanoparticles loaded with GFP (green florescent protein)-encoding plasmid DNA were conjugated to bacteria via biotinylated antibody and antigen interactions. Such conjugation resulted in plasmid survival when faced with the acidic endosomal environment and intracellular enzymes. As a result, this hybrid delivery system exhibited excellent tumor enrichment and achieved 380-fold enhancement of gene expression compared to a mock control group.

Taken together, bacteria can elevate the enrichment of nanoparticles at tumor lesions and facilitate the delivery of cargos to the appropriate subcellular location. Nanoparticles enhance the drug-carrying ability of bacterial vectors. Bacteria–nanoparticle hybrid delivery systems have the unique advantage of effective cancer-targeting ability, efficient drug loading, and proven subcellular delivery. However, the impact of nanoparticle contents on the bacterium’s tumor-targeting ability needs to be well-understood. In general, the fewer the loaded nanoparticles, the lesser the impact on their tumor-targeting capability. In addition, the influence of the conjugation method for the bacteria and nanoparticles on their drug target delivery performance needs to be examined. For example, different conjugation methods like electrostatic adsorption, physical attachment, antibody–antigen specific interaction may result in different stability in the physiological environment, thus influencing their on-target drug delivery.

#### 4.2.2. Other Functional Properties

Apart from acting as drug carriers, nanoparticles can also act as active compounds which endow a bacterial therapy conducive to diverse treatments, such as photothermal therapy and enzyme-like therapy. In this section, we introduced several innovative therapies realized through such hybrid systems.

**Figure 5:**
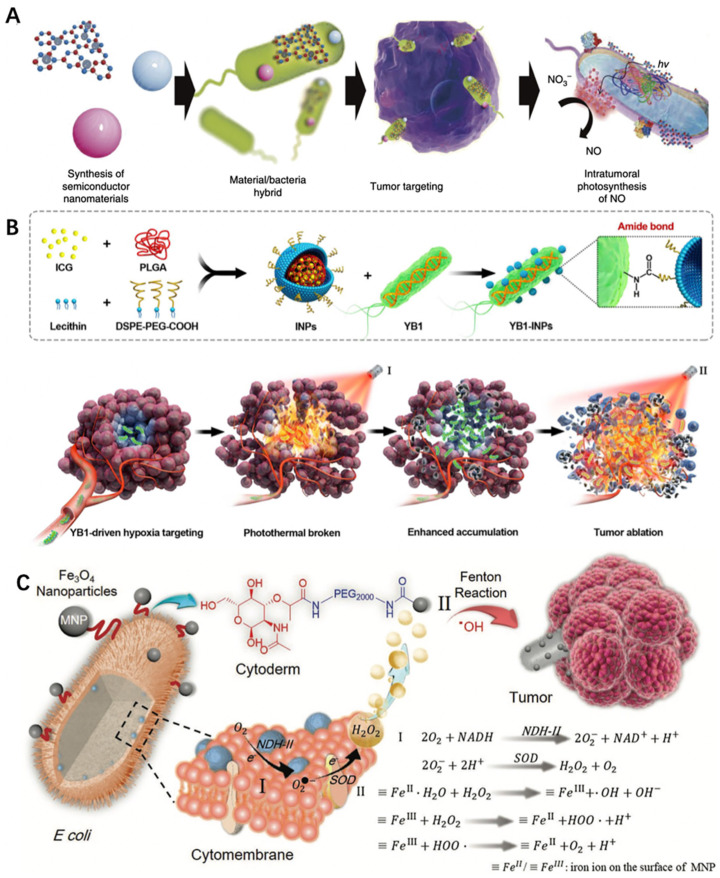
(**A**) Schematic diagram of photo-controlled bacterial therapy. Reproduced from [85], 2018, Springer Nature. (**B**) Preparation procedure of YB1–INPs. Synthesized INPs with single-step sonication were attached to YB1 through amide bonds. YB1–INPs with hypoxia-targeting and photothermal-assisted bioaccumulation for tumor penetrative therapy. After migration into tumor hypoxic cores and subsequent irradiation with a NIR laser, the loosening of the tumor tissue and tumor lysis generate bacteria-attracting nutrients, which further enhances the accumulation and coverage of YB1–INPs in large solid tumors. Ultimately, the enriched YB1–INPs under NIR laser irradiation completely ablated the large solid tumor without relapse. Reproduced with permission from [86], 2019, Elsevier. (**C**) The scheme of a bacteria-based Fenton-like bioreactor and its chemodynamic therapy process for antitumor therapy. Reproduced with permission from [87], 2019, WILEY-VCH Verlag GmbH & Co. KGaA, Weinheim.

Photocatalytic therapy takes effect through a dual sequential strategy, in which photosensitizers are first enriched at the target tissue and then the light is applied to activate the agents [84]. Based on this scheme, Zheng et al. charged bacteria with a nanophotocatalyst for photo-controlled bacterial therapy [85]. In their study, carbon nitride (C_3_N_4_) was combined with *E. coli* through electrostatic attraction. Upon light irradiation, photoelectrons produced by C_3_N_4_ flowed into *E. coli* to enhance the enzymatic reduction of endogenous NO^3−^ into toxic NO (Figure 5A). As a result, such combinational therapy greatly improved the therapeutic outcome and achieved approximately 80% tumor regression (*E. coli* alone only achieved ~20% tumor regression).

Photothermal therapy (PTT) utilizes a photothermal sensitizing agent to convert light energy into heat and induces tumor regression [88]. Chen et al. recently developed nanophotosensitizer-engineered *Salmonella* bacteria to treat cancer [86]. In their study, nanophotosensitizers (indocyanine green (ICG)-loaded nanoparticles) were conjugated to YB1, a genetically modified and safe *Salmonella typhimurium* strain, via amide bond conjugation (YB1–INPs). After intravenous injection and tumor accumulation of YB1–INP, near-infrared (NIR) light was first applied to lyse the tumor cells. This loosened tumor tissues and released bacteria-attracting nutrients that further enhanced the bacterial enrichment in the cancer tissue. At that time, the second NIR irradiation was applied to completely eradicate the established solid tumor without relapse (Figure 5B).

Nanomaterials can also serve as an efficient catalyst of specific chemical processes due to their own intrinsic properties without the need of external activation, as is the case of metal-based nanoparticles, e.g., ceria nanoparticles and iron oxide nanoparticles [89,90,91]. Such enzyme-like properties can be combined with bacterial therapy to treat cancer [92]. For example, Fan et al. recently innovated a bacteria-based Fenton-like reaction bioreactor [87]. In their study, *Escherichia coli* MG1655 was bioengineered to overexpress respiratory chain enzyme II (NDH-2), which remarkably elevated the H_2_O_2_ concentration in tumor tissues. Magnetic iron oxide nanoparticles were covalently conjugated to the surface of bacteria which catalyzed excessive H_2_O_2_ to toxic hydroxyl radicals (Figure 5C). As a result, such bacteria–nanoparticle hybrid systems exhibited outstanding tumor colonization and self-supplied Fenton-like reactions, producing a strong inhibitory effect on tumor growth in a CT26 tumor-bearing mouse model.

Taken together, nanomaterials provide a promising collection of largely underdeveloped therapeutic tools that can be applied to bacterial therapy for cancer treatment due to their diverse attractive physical and chemical properties (Table 3). However, difficulties and opportunities coexist in this area. As mentioned above, photocatalytic therapy, photothermal therapy, and nanomaterial-based catalytic therapy are three typical additional treatments. The applied light intensity and photoperiod need to be optimized for better therapeutic efficacy. In addition, the enzyme properties of nanomaterials are relatively unstable in different physiological environments which influences the repeatability of such hybrid platforms’ therapeutic efficacy.

## 5. Conclusions and Prospects

In this review, we outlined the therapeutic roles of nanotechnology-facilitated bacteria-based drug and gene delivery systems. With the help of nanotechnology, such hybrid systems exhibit strong capabilities of delivering drugs and genetic information to targeted tumor sites at high specificity for precise subcellular locations. In addition, nanomaterials can serve as active pharmaceutic compounds by themselves or when hybridized with bacteria and bring additional therapeutic potential to bacterial therapy, including proven techniques such as photothermal or catalytic combinational treatment. As a result, such hybridization exhibits no noticeable impact on bacterial targeting of tumor tissues and exerts great synergistic therapeutic efficacy against cancer. On the other hand, the appropriate selection of bacteria is critical for improving drug-targeting ability. For example, Felfoul et al. conjugated drug-loaded liposomes to *Magnetococcus marinus* strain MC-1 [96]. As a result, up to 55% of the MC-1 cells penetrated hypoxic regions of HCT116 colorectal xenografts when injected near the tumor with the aid of external magnetic forces. Such hybrid drug delivery systems can significantly improve the therapeutic index of various small-molecule drugs in the tumor’s hypoxic regions. Therefore, the rational design of bacteria–nanoparticle hybrid systems is essential to achieve their full potential performance for treating cancer.

Despite great achievements and promising outlooks in this area, several critical issues must be solved before their possible translation into clinical use. Firstly, latent inflammation and toxicity induced by bacterial membrane components need to be well-managed to avoid severe systemic inflammation [97]. Secondly, therapeutic efficacy and replicability need to be verified carefully in trials. Many variables, such as the amounts of bacteria, nanoparticles, drugs, and genetic information, and the method of nanoparticle hybridization with bacteria must be carefully considered during the construction of such hybrid systems. Overall, nanotechnology has unlocked a new era for bacteria-based cancer therapy and will bring benefits to clinical cancer treatment in new, innovative ways.

## Figures and Tables

**Table 1:** Timeline of several typical examples of bacteria use in cancer treatment.

Year	Bacteria	Cancer Type	Brief Description	Ref.
1868	*Streptococcus pyogen* *es*	Sarcoma	First use of bacteria in cancer treatment	[20]
1891	*Streptococcus pyogen* *es*	Malignant sarcoma	Coley’s toxins	[21]
1989	*Mycobacterium bovis*	Bladder cancer	Bacillus Calmette–Guerin vaccine (BCG) approved by the FDA	[22]
2000	*Salmonella typhimurium* VNP20009	Solid tumor	Deletion of the *pur*I and *msb*B genes which reduce the virulence and the risk of septic shock	[23]
2005	*Clostridium novyi*-NT	HCT116 colorectal cancer	Combination of bacterial therapy and traditional drug therapy	[24]
2006	*Escherichia coli*	HeLa, HepG2, and U2OS cell lines	Characterization of invasin from *Yersinia pseudotuberculosis* as an output module	[25]
2011	*Salmonella Typhimurium* SL7207	Colorectal carcinoma	Engineered to survive only in anaerobic conditions without otherwise affecting its functions	[26]

**Table 2:** Bacteria membrane-based nanoparticles in cancer treatment.

Membrane Source	Cancer Type	Membrane Type	Cargo	Efficacy	Ref.
*Salmonella*	B16F10 and 4T1 tumors	OMV	Tegafur@F127 nanomicelles	(1) Surface is modified with RGD to preferentially accumulate in tumor tissues (2) Combination of chemotherapy and immunotherapy	[71]
	Ehrlich ascites carcinoma (EAC)	OMV	Paclitaxel	(1) Passive accumulation in tumor tissues through the EPR effect (2) Combination of chemotherapy and immunotherapy	[72]
*Escherichia coli*	Human lung carcinoma A459 cells	Protoplast-derived nanovesicles	Doxorubicin	(1) Bioengineered with high expression of the epidermal growth factor to target the tumor (2) Alleviation of systemic toxicity of the chemotherapeutic agent	[73]
	B16F10 tumor	DMV	Doxorubicin	(1) Bioengineered with high expression of RGD motifs to target the tumor (2) Targeting of the neutrophils or monocytes that mediate transportation towards the tumor	[74]
	HER2-overexpressing HCC1954 cells	OMV	siRNA	(1) Targeting of tumor tissues via the EPR effect (2) Avoidance of gene leakage and protection from degradation	[75]
	CT26 and 4T1 tumors	OMV	ICG	(1) Surface is functionalized with a calcium phosphate shell to respond to the acidic environment of the tumor (2) Combination of photothermal therapy and immunotherapy	[68]
	B16F10 tumor	OMV	ICG	(1) Transdermal nanoplatform against melanoma (2) Combination of photothermal, photodynamic therapy, and immunotherapy	[76]
	TC-1 and B16F10 tumors	OMV	BFGF	(1) Use as a cancer vaccine (2) Induction of production of the antibodies that target tumor angiogenesis	[77]

Abbreviations: RGD: amino sequence of arginine, glycine, and aspartate; ICG: indocyanine green; BFGF: basic fibroblast growth factor.

**Table 3:** Bacteria–nanoparticle hybrid systems in cancer treatment.

Bacterium	Cancer Type	Nanoparticle	Cargo	Efficacy/Therapeutic Mechanism	Ref.
*S. typhimurium* VNP20009	4T1 tumor	PLGA	**/**	Remarkable (up to 100-fold) enhancement of nanoparticle retention and distribution in solid tumors	[78]
*Bifidobacterium longum*	MDA-MB-231 breast tumor	PLGA	Low-boiling-point perfluorohexane (PFH)	Combination of diagnostic and therapeutic efficacyRealization of high-intensity focused ultrasound therapy against cancer	[79]
*L. monocytogenes*	MCF-7, HT29, KB, HepG-2 cancer cells	Polystyrene nanoparticles	GFP-encoding plasmid DNA	High resistance toward the acidic endosome environment and intracellular enzymes and successful delivery of genes into the nucleus	[83]
*Escherichia coli*	4T1 and CT26 tumors	Carbon nitride (C_3_N_4_) semiconductor nanomaterials	/	Achievement of approximately 80% tumor regression superior than with *E. coli* alone (~20%)	[85]
*Salmonella typhimurium* YB1	MB49 tumor	PLGA	ICG	Highly efficient photothermal ability to eradicate established solid tumors without relapse	[86]
*Escherichia coli* MG1655	CT26 tumor	Magnetic Fe_3_O_4_ nanoparticles	/	Achievement of effective tumor colonization and realization of a self-supplied therapeutic Fenton-like reaction to cure cancer without an additional H_2_O_2_ source	[87]
*Escherichia coli*	HOS, MG63, and U2OS cancer cells	Polydopamine nanoparticles	Ce6	An ability to provide catalase and convert endogenic hydrogen peroxide into oxygen for subsequent photodynamic therapy	[93]
*Shewanella oneidensis* MR-1	CT26 tumor	Manganese dioxide nanoflowers	/	MnO_2_ serves as electron acceptor, tumor metabolite lactic acid performs as an electron donor, resulting in continuous consumption of lactic acid in cancer cells	[94]
*Synechococcus* 7942	4T1 tumor	Human serum albumin nanoparticles	ICG	In situ photocatalyzed oxygen generation enabling robust immunogenic PDT against tumor growth and metastasis	[95]

## Data Availability

Not applicable.

## Sample Availability

Samples of the compounds are not available from the authors.
